# Asynchronous BCI control using high-frequency SSVEP

**DOI:** 10.1186/1743-0003-8-39

**Published:** 2011-07-14

**Authors:** Pablo F Diez, Vicente A Mut, Enrique M Avila Perona, Eric Laciar Leber

**Affiliations:** 1 Gabinete de Tecnología Médica (GATEME), Facultad de Ingeniería, Universidad Nacional de San Juan, San Juan, Argentina; 2 Instituto de Automática (INAUT), Facultad de Ingeniería, Universidad Nacional de San Juan, San Juan, Argentina

## Abstract

**Background:**

Steady-State Visual Evoked Potential (SSVEP) is a visual cortical response evoked by repetitive stimuli with a light source flickering at frequencies above 4 Hz and could be classified into three ranges: low (up to 12 Hz), medium (12-30) and high frequency (> 30 Hz). SSVEP-based Brain-Computer Interfaces (BCI) are principally focused on the low and medium range of frequencies whereas there are only a few projects in the high-frequency range. However, they only evaluate the performance of different methods to extract SSVEP.

**Methods:**

This research proposed a high-frequency SSVEP-based asynchronous BCI in order to control the navigation of a mobile object on the screen through a scenario and to reach its final destination. This could help impaired people to navigate a robotic wheelchair. There were three different scenarios with different difficulty levels (easy, medium and difficult). The signal processing method is based on Fourier transform and three EEG measurement channels.

**Results:**

The research obtained accuracies ranging in classification from 65% to 100% with Information Transfer Rate varying from 9.4 to 45 bits/min.

**Conclusions:**

Our proposed method allows all subjects participating in the study to control the mobile object and to reach a final target without prior training.

## Background

A Brain-Computer Interface (BCI) is a system that helps impaired people to control a device (such as a robotic wheelchair) using their own brain signals. These brain signals can be obtained from the scalp as electroencephalographic (EEG) signals.

A Steady-State Visual Evoked Potential (SSVEP) is a resonance phenomenon arising mainly in the visual cortex when a person is focusing the visual attention on a light source flickering with a frequency above 4 Hz [1]. SSVEPs are periodic, with a stationary distinct spectrum showing characteristic SSVEPs peaks, stable over time [2].

The SSVEP can be elicited up to at least 90 Hz [3] and could be classified into three ranges: low (up to 12 Hz), medium (12-30) and high frequency (> 30 Hz) [1]. In general, the SSVEP in low frequency range has larger amplitude responses than in the medium range. Consequently, while the larger the amplitude of the SSVEP, the easier its detection. The weakest SSVEP is found in the high frequency range. However, spontaneous EEG (considered here as noise) decrease in higher frequency bands, hence, the signal to noise ratio is similar for three ranges [4].

However, the majority of SSVEP-based BCI are principally focused in the low and medium range of frequencies [5-8]. There is only scant research in the high frequency range: in [9] Independent Component Analysis (ICA) was used to detect early SSVEP at 8.8 and 35 Hz, in [10] an alternate half field SSVEP is implemented between 25 to 40 Hz for the detection of 8 symbols on a virtual keypad. Canonical correlation analysis (CCA) is applied to detect SSVEP in the 27 to 43 Hz range in [11]. In [12] the Wavelet Transform and Hilbert-Huang Transform (HHT) are compared to detect SSVEP in 10 s EEG for stimulation between 30 up to 50 Hz. More recently, spatial filters were applied to enhance the SSVEP detection in four oscillatory visual stimuli at 30, 35, 40, and 45 Hz, in [13].

In [9] and [11-13] off-line analysis of the EEG were performed using methods with medium to high computational cost such as ICA, CCA, HHT and spatial filters. An updated and interesting review of SSVEP-based BCI is presented in [14], where frequencies, stimulation devices, colour, bit rate and other details of BCIs are offered.

The high-frequency SSVEP range has the advantage of a great decrease of visual fatigue caused by flickering [10,12,15], making the SSVEP-based BCI a more comfortable and stable system [15]. Besides, low and medium frequency SSVEP ranges interfere with alpha rhythm, and could cause an epileptic seizure as well [16].

Finally, a BCI can be classified into synchronous or asynchronous. A synchronous BCI needs a synchronization cue for the beginning of each mental task (or gazing at a flickering light), i.e., it is a time-locked BCI. On the other hand, in asynchronous BCI the ongoing EEG is used since the subject can change his mental state (or gaze at a light) at any moment. Of course, asynchronous BCI is more difficult to implement, since they can experience idle states where the user does not gaze at any flickering light.

The objective of this research is to control the navigation of a mobile object on the screen through different environments using a high-frequency SSVEP-based asynchronous BCI. A future objective of this approach aims at the navigation of a mobile robot (e.g. a robotic wheelchair) under partially-structured environments.

## Methods

### EEG acquisition

**Figure 1:**
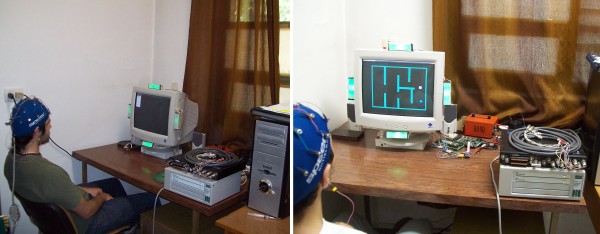
**EEG acquisition equipment and lights on the sides of monitor**. Left image: A subject using the SSVEP-based BCI. Right image: acquisition equipment and monitor displaying the difficult scenario.

Six subjects (ages 32 ± 3; 1 F and 5 M) participated in this study. All subjects provided written consent to participate and ethical approval was granted by the institutional ethics committee. The subjects were seated in a comfortable chair in front of a monitor with four bars on each side (10 cm × 2.5 cm), illuminated by high efficiency light-emitting diodes (LEDs) (Figure 1). These LEDs are flicker at 37, 38, 39 and 40 Hz for the bars on top, to the right, then down and to the bars on the left, respectively. These flickering frequencies are almost unperceivable by the user. The frequency of each LED is precisely controlled with an FPGA Xilinx Spartan2E.

The EEG was measured with six channels at O1, Oz, O2, P3, Pz and P4, referenced to FZ and grounded at linked A1-A2, but only O1, Oz and O2 channels were used for on-line feedback. These positions were chosen since they are over visual cortex where SSVEP have higher amplitude [2]. Positions P3, Pz and P4 were acquired for further studies, but they were not used in this work. The EEG signals were acquired with a Grass MP15 amplifiers system and digitalized with a NI-DAQPad6015 (Sample Frequency = 256 Hz for each channel). Cut-off frequencies of analogical pass-band filter were set to 3 and 100 Hz and a notch filter for 50 Hz line interference was used.

For each subject, a baseline EEG was acquired previous to the experiment, where the subjects were asked to focus on a point in the centre of the screen for 60 s, but not to focus on any bar. This baseline was used for equalization of EEG spectrum; this will be explained on Section 3. Two different experiments were carried out: 1) A time-locked (synchronous) step and 2) An asynchronous control step.

### Time-locked step

The purpose of this step is to evaluate the performance of the proposed interface in a controlled experiment, since in the next step (asynchronous control) the subject is who controls the experiment. For this purpose, this step is divided into trials, where, the light that the subject must gaze at is indicated for each trial.

Each trial lasted 10 s with a variable separation between trials from 2 to 4 s. The trial begins with a beep (t = 0 s) and 2 s later a flickering bar is randomly indicated to the subject with an arrow on the screen; at this time the EEG signal is processed on-line and feedback is presented at the end of each trial. All subjects participated in four sessions and each session contains 20 trials, with only a few minutes between sessions.

The possible results of the classification process were:

1. Correct: an SSVEP was detected and it corresponds to the bar indicated by the arrow on the screen, this is a True Positive (TP).

2. Incorrect: an SSVEP was detected and it is different from the bar indicated by the arrow on the screen, this is a False Positive (FP).

3. No detection: this situation occurs when the subject does not concentrate enough on the light or the proposed method does not detect an SSVEP, this is a False Negative (FN).

Additional file 1 shows a subject performing this time-locked step.

### Asynchronous control step

**Figure 2:**
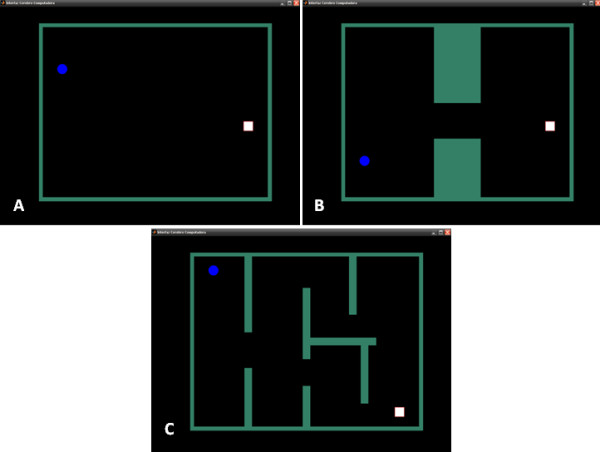
**Different scenarios proposed for ongoing EEG**. (easy, medium and difficult scenarios). Blue circle: the ball; white square: final spot.

In order to evaluate the performance of the proposed method for ongoing EEG, software was developed where the user had to control a mobile ball and navigate it through a scenario to reach a final spot (white square). There were three different scenarios with different difficulty levels (easy, medium and difficult) as can be seen in Figure 2. The user can choose his path to reach the final destination.

When a SSVEP is detected the ball moves in the direction of the detected light, and it continues moving until another SSVEP is detected or when the ball hits a wall. The user can stop the ball gazing at the opposite light of the current direction. The experiment ends when mobile ball arrives to the final spot or when more than 3 minutes are required to complete the task. Additional file 2 shows some subjects navigating the ball through the three scenarios.

### EEG signal processing

The EEG was analysed with a window of 2 s duration, moving in steps of 0.25 s, i.e., the EEG signal processing is performed 4 times by second. The processing method is similar to a previous research project done by our group [17], this one was based in [7] (but they were applied to detection of medium and low frequencies SSVEP).

A Butterworth band-pass digital filter, order 6, with 32 and 45 Hz cut-off frequencies was utilized. Afterwards, the periodogram was computed. It is an estimation of the power spectral density based on the Discrete Time Fourier Transform (DTFT) of the signal x[n]defined as:(1)

where *S_P _(f) *is the periodogram, *T_S _*is the sampling period, *N *is the number of samples of the signal and *f *is the frequency. To compute the periodogram, the Fast Fourier Transform (FFT) with 2 s length rectangular window and zero padding to 1024 points was used.

Following that, we propose to compute the normalized power at each stimulation frequency as the mean value of the power on each channel [17]:(2)

where *P(fi) *is the normalized power estimation for frequency *fi *(i = 37, 38, 39 or 40 Hz); *ch *is the number of channel; *Δf *is the bandwidth of the power estimation: ± 0.25 Hz;  is the periodogram of baseline EEG used for equalization purpose, since the EEG spectrum has lower power for higher frequencies. This means that these values vary depending on their frequency range. For example, an SSVEP at 37 Hz has larger amplitude than another SSVEP at 40 Hz. In order to compute *P(fi)*, O1, Oz and O2 channels were used, consequently *M *= 3. This calculation was performed every 0.25 s.

An SSVEP is labelled as one of the four possible classes (top, right, down or left) if the maximum *P(fi) *is maintained for a determined period of time *H*:(3)

The time-threshold *H *in time-locked (synchronous) step is *H_S _*and fixed at 1.75 s for on-line feedback. In asynchronous step *H_A _*can be adjusted from 1.5 s to 2.25 s.

## Results

Table 1 shows the results in time-locked step for different *H_S _*values and are detailed the Correct, Incorrect and Non-detected trials, average time by trial and the Information Transfer Rate (ITR). The ITR is a measure of the information transmitted and is calculated as [18]:(4)

**Table 1:** Results in time-locked step

**Subject**	**Hs = 1,5 s**						**Hs = 1,75 s**						**Hs = 2 s**						**Hs = 2,25 s**					
				
	**TP**	**FP**	**FN**	**Time [s]**	**bits/trial**	**bits/min**	**TP**	**FP**	**FN**	**Time [s]**	**bits/trial**	**bits/min**	**TP**	**FP**	**FN**	**Time [s]**	**bits/trial**	**bits/min**	**TP**	**FP**	**FN**	**Time [s]**	**bits/trial**	**bits/min**
			
**1**	**100**	0	0	2.66 ± 0.36	2	**45.1**	**100**	0	0	2.91 ± 0.36	2	41.2	**100**	0	0	3.16 ± 0.36	2	38	**100**	0	0	3.41 ± 0.36	2	35.2
**2**	**98.8**	1.25	0	2.84 ± 0.69	1.89	**39.9**	**98.8**	0	1.25	3.09 ± 0.69	1.98	38.5	**98.8**	0	1.25	3.43 ± 0.88	1.98	34.7	97.5	0	2,5	3.65 ± 0.77	1.8	29.9
**3**	80	18.8	1.3	3.11 ± 0.8	0.99	19.20	81.3	13.8	5	3.44 ± 0.95	1.14	19.99	81.3	7.5	11.3	3.86 ± 1.12	1.33	20.7	**82.5**	3.8	13.8	4.12 ± 1.12	1.47	**21.50**
**4**	**78.8**	21.3	0.0	2.98 ± 0.96	0.92	18.5	77.5	17.5	5.0	3.52 ± 1.37	0.92	15.7	72.5	15	12.5	3.74 ± 1.32	1	16.2	66.3	10	23.8	3.98 ± 1.30	**1.04**	15.8
**5**	56.3	40	3.8	4.02 ± 1.39	0.38	5.70	62.5	26.3	11.3	4.67 ± 1.44	0.67	8.60	**65**	15	20	5.14 ± 1.55	0.92	**10.76**	57.5	10	32.5	5.36 ± 1.55	0.92	10.37
**6**	**65**	31.3	3.8	3.83 ± 1.34	0.59	9.18	62.5	27.5	10	4.29 ± 1.41	0.64	9	53.8	17.5	28.8	4.77 ± 1.48	0.75	**9.42**	45	10	45	5.07 ± 1.53	0.75	8.93

The values represent the percentages of True Positive (TP), False Positive (FP), and False Negative (FN), the average time (mean ± std) by trial and the ITR in bits/trial and in bits/minute, evaluated for different *H_S_*. In bold: the best results per subject.

where *P_r _*is the probability of non-detected cases, *P_w _*is the probability of incorrect detected cases and *N *is the number of targets (in our case N = 4). The ITR could be expresed in bits/trial or in bits/min.

**Figure 3:**
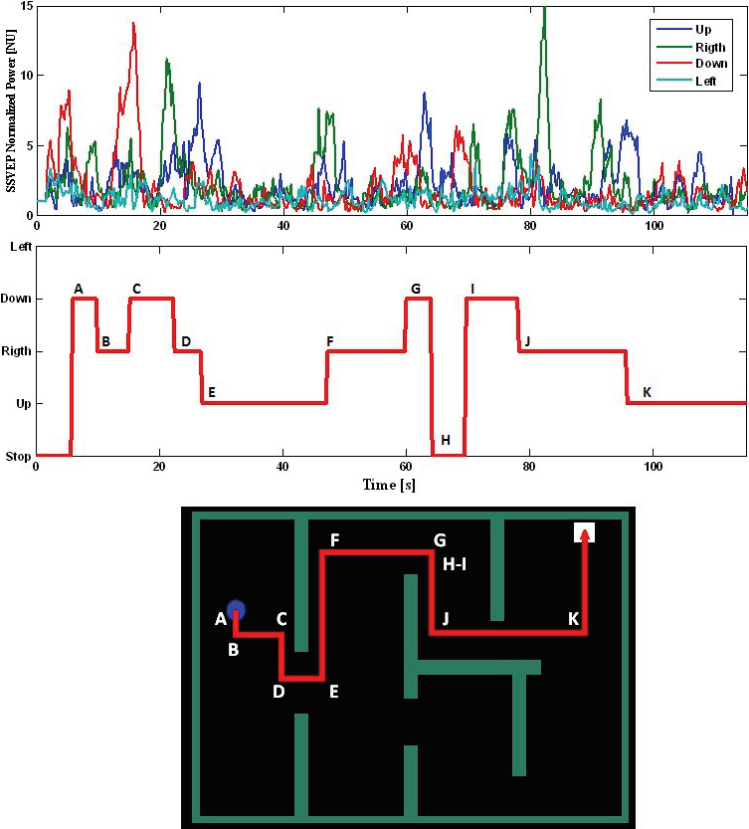
**A trial in the hard scenario**. (a) power calculated on-line, (b) Direction changes (c) Path followed through the scenario. The direction changes are marked by letters. In t = 64 the ball stops due to a FP (H point).

In asynchromous mode, the SSVEP-power calculated on-line for Subject 5 is presented in Figure 3a. The SSVEP-power increases when the subject gazes at a determined ligth and SSVEP-power is labeled as a class when time-threshold *H_A _*is overcome, i.e., the ball changes its movement. In this case *H_A _*was 2.25 s.

Figure 3b shows the direction changes along the task. In t = 64 s the ball stops since the top-ligth is detected at this moment (the contrary to current direction), this detection was considered as a FP. Threshold *H_A _*was adjusted for each subject in order to reject FP, although some FP were detected anyway, but adjusting to optimal *H_A _*the FP rate was lower. Figure 3c shows the path followed by the ball to reach the final spot (white square).

Table 2 presents the results for each subject moving the ball in difficult scenarios. This table, details the mean and standard deviation values of the task time and the number of decisions made to accomplish the task and the TP and FP (and its percentages). The last column on this Table is the number of times that the subject performs the task and when he/she reaches the final destination. It shows the best *H_A _*for each subject as well. Finally, when subject is gazing the centre of the screen (looking the moving ball) most of the time, no classes (top, right, left or down) should be detected. Occasionally, if an SSVEP is detected in this situation, it is considered as a FP (see Tables 1 and 2).

**Table 2:** Average values on difficult scenario

Subjects	H_A_		Task Time [s]	Decisions/task			Reach final destination?
					
				Total	TP	FP	
**1**	**2**	**Average**	96.2	12.2	11.6	0.6	100%
		**Std. Dev**.	12	2.8	2.1	0.9	(5 of 5)
		**%**			**95.1**	**4.9**	

**2**	**2**	**Average**	129.8	19.8	18.3	1.5	100%
		**Std. Dev**.	9.4	4	3.4	1.3	(4 of 4)
		**%**			**92.4**	**7.6**	

**3**	**2**	**Average**	108.3	15.7	14.3	1.3	100%
		**Std. Dev**.	37.4	5.5	4.5	1.2	(3 of 3)
		**%**			**91.5**	**8.5**	

**4**	**2.25**	**Average**	161.3	18.3	15	3.3	67%
		**Std. Dev**.	22.8	3.1	2.7	0.6	(2 of 3)
		**%**			**82.8**	**18.2**	

**5**	**2.25**	**Average**	149	16.3	13.7	2.7	67%
		**Std. Dev**.	31.5	4.7	3.2	1.5	(2 of 3)
		**%**			**83.7**	**16.3**	

**6**	**2.25**	**Average**	176	20.3	15	5.3	100%
		**Std. Dev**.	2.6	6.7	4.6	2.1	(3 of 3)
		**%**			**73.8**	**26.2**	

## Discussion

Ongoing EEG classification of SSVEP (no high-frequency) based-BCI is implemented in [6,7] using a refractory time (when no decisions are allowed), in order to control grasping with a robotic arm. This refractory time is implemented to avoid FP since the robotic arm takes a time to perform each movement. In our case the subjects can make decisions every time they want to and the FP are avoided (or diminished) by adjusting the time-threshold *H*.

Other methods used for high-frequency SSVEP detection are more expensive computationally [9,11-13], and they are evaluated in off-line analysis of the EEG but they were not evaluated for asynchronous EEG classification. Using spatial filters, an ITR of 22.7 bits/min was reached in [13].

A method to control a mobile robot in indoor environments was presented in [18], but the subjects need a few days of training to control the mobile robot. In this case, the subject can control the mobile object on the screen in only a few minutes. This is an advantage of SSVEP based-BCI over other kinds of BCI.

The proposed method achieves accuracy in classification ranging from 65% to 100%. This could be translated into ITR ranging from 9.4 to 45 bits/min. A high bit rate is not required to control a mobile object, since it is not necessary to make decisions every second, e.g., when it navigates through a corridor. Hence the ITR achieved in this research is more than enough to control a mobile object. This is claimed since the subjects can almost always effectively navigate the mobile ball to the final spot most of the time.

In locked-time step, for lower time-threshold *H_S _*higher wrong cases were obtained (Table 1); when *H_S _*is increased these wrong cases were evaluated as non-detected whereas correct cases were not as detrimental. Therefore, adjusting *H_S _*is possible to reduce the wrongly-detected cases and to obtain similar accuracy in detection of SSVEP. If *H_S _*parameter overcome a certain value (depending on each subject) it will eventually be unable to detect a class (top, right, down or left) because it is more difficult maintain a SSVEP for long periods of time.

In asynchronous mode, easy and medium scenarios were used to adjust the time-threshold *H_A_*, and then the performance of each subject was evaluated in the difficult scenario. Besides, in both of these scenarios the subject learns how to control the ball since it is a hard task, i.e., when to gaze at the light in order to change the movement of the ball at the right time and avoid hitting a wall. For this purpose, those scenarios were repeated a few times (no more than 5 ± 2 in average), depending on the Subject performance.

Moreover, sometimes the subjects did not want to convey any command to the moving ball, however lights are still in their visual field and a command could be detected and transmitted to the ball. This problem is called the "Midas Touch Effect" [19] and this is the reason for the FP. This effect became evident when the moving ball was navigated close to the sides of the screen where the lights are located.

In order to mitigate this effect an adjustable time-threshold *H_A _*was implemented. With short time-threshold more FP were attained and the navigation of the ball became unstable. On the other hand, with long time-threshold *H_A _*less FP were attained but it was harder to change the movement. Hence, for each subject the time-threshold was adjusted in order to obtain a comfortable navigation of the ball. The time-threshold was adjusted from 1.75 s up to 2.25 s, depending on the subject. The threshold *H_A _*in Table 2 is not necessarily the same *H_S _*that allows the best ITR in Table 1, since they are evaluated under different experimental conditions. In Table 1, the experiment is in synchronous mode, whereas in Table 2, the experiment is in asynchronous mode and the threshold *H_A _*is adjusted in order to get a comfortable navigation of the ball (avoiding, as much as possible, the Midas touch effect).

The threshold *H_A _*in asynchronous step was 2 or 2.25 s (see Table 2), hence *H_A _*could be used in a fixed value of 2.25 s (the subjects with *H_A _*= 2 s could navigate the ball with *H_A _*= 2.25 s without performance detriments). However, always is advisable to adapt the BCI in order to attain the optimum performance.

Once the time-threshold was adjusted, subjects had to control the ball in the difficult scenario and navigate it to the final spot. They accomplished this work in almost all cases (except one time in Subjects 4 and 5). The subjects who obtained low ITR in the time-locked step accomplished the work too, however they needed more time than subjects with high ITR.

All subjects participated in this study were asked about discomfort with flickering, no one express discomfort. According with others studies [10,12,15], we observe that high-frequencies SSVEP produce much less visual fatigue than lower frequencies. Furthermore, the discomfort of subjects observed in a previous work of our group [17], using SSVEP in medium frequency range (13 to 16 Hz), was less compared to this work.

In summary, the asynchronous BCI proposed in this work allows the effective control of a mobile object on the screen with high-frequency SSVEP (which are less annoying) and using a simple method to extract SSVEP from ongoing EEG.

## Conclusions

In this work, an asynchronous BCI based in high-frequency SSVEP is presented, using only three ongoing EEG channels in order to control a mobile object on the screen. Besides, it used a simple method to detect the SSVEP, i.e., mean powers of each stimulation frequency evaluated on the periodogram. It obtained accurate classification among 65% to 100% with ITR ranging from 9.4 to 45 bits/min.

This method allows to all subjects participating in the study to control the mobile object and to reach a final target without training, by only adjusting one parameter, the time-threshold *H*. Furthermore, impaired people could be benefit from this method since it could be easily extended to control a robotic wheelchair.

Written informed consent was obtained for publication of this case report and accompanying images. A copy of the written consent is available for review by the Editor-in-Chief of this journal.

## Competing interests

The authors declare that they have no competing interests.

## Authors' contributions

PFD wrote the algorithms and performed the experiments with help from EAP. VAM and ELL contributed with initial ideas and advisory. All authors reviewed and approved the final manuscript.

## Supplementary Material

Additional file 1 **BCI Synchronous step**. A movie shows the BCI time-locked (synchronous) step. Click here for file

Additional file 2 **BCI asynchronous step**. Another movie shows the BCI asynchronous step control of mobile object on the screen through different scenarios. Click here for file
